# Association of Small HDL Subclasses with Mortality Risk in Chronic Kidney Disease

**DOI:** 10.3390/antiox13121511

**Published:** 2024-12-11

**Authors:** Julia T. Stadler, Andrea Borenich, Anja Pammer, Insa E. Emrich, Hansjörg Habisch, Tobias Madl, Gunnar H. Heine, Gunther Marsche

**Affiliations:** 1 Division of Pharmacology, Otto Loewi Research Center for Vascular Biology, Immunology and Inflammation, Medical University of Graz, Neue Stiftingtalstraße 6, 8010 Graz, Austria; julia.stadler@medunigraz.at (J.T.S.); anja.pammer@medunigraz.at (A.P.); 2 Institute of Pharmaceutical Sciences, Department of Pharmacognosy, University of Graz, Beethovenstraße 8, 8010 Graz, Austria; 3 Institute for Medical Informatics, Statistics and Documentation, Medical University of Graz, Auenbruggerplatz 2, 8036 Graz, Austria; andrea.borenich@medunigraz.at; 4 Faculty of Medicine, Saarland University, 66421 Saarbrücken, Germany; insa.emrich@uks.eu; 5 Division of Medical Chemistry, Otto Loewi Research Center for Vascular Biology, Immunology and Inflammation, Medical University of Graz, Neue Stiftingtalstraße 6, 8010 Graz, Austria; hansjoerg.habisch@medunigraz.at (H.H.); tobias.madl@medunigraz.at (T.M.); 6 BioTechMed Graz, 8010 Graz, Austria; 7 Department of Nephrology, Agaplesion Markus Krankenhaus, 60431 Frankfurt am Main, Germany

**Keywords:** HDL subclasses, chronic kidney disease, survival, lipoproteins, mortality, apolipoproteins

## Abstract

High-density lipoproteins (HDL) exist in various subclasses, with smaller HDL particles possessing the highest anti-oxidative and anti-inflammatory properties. Understanding the role of these specific subclasses in chronic kidney disease (CKD) could provide valuable insights into disease progression and potential therapeutic targets. In the present study, we assessed HDL subclass composition in 463 patients with CKD stage 2–4 using nuclear magnetic resonance spectroscopy. Over a mean follow-up period of 5.0 years, 18.6% of patients died. Compared to survivors, deceased patients exhibited significantly lower levels of cholesterol, ApoA-I, and ApoA-II within the small and extra-small (XS) HDL subclasses. Multivariable Cox regression analysis, adjusted for traditional cardiovascular and renal risk factors, demonstrated that reduced levels of XS-HDL-cholesterol, XS-HDL-ApoA-I, and XS-HDL-ApoA-II were independently associated with an increased risk of mortality. Furthermore, receiver operating characteristic analysis identified XS-HDL-ApoA-II as the most potent prognostic marker for mortality. In conclusion, reduced small and XS-HDL subclasses, especially XS-HDL-ApoA-II, are strongly associated with increased all-cause mortality risk in CKD patients. Assessment of HDL subclass distribution could provide valuable clinical information and help identify patients at high risk.

## 1. Introduction

Chronic kidney disease (CKD) is a significant global health concern associated with a multitude of complications, including cardiovascular disease and accelerated aging [1,2]. Beyond traditional risk factors, CKD patients often exhibit biochemical abnormalities like endothelial dysfunction and inflammation, which contribute to increased morbidity and mortality [3,4,5,6]. Early detection and comprehensive management are crucial to mitigate these risks and improve patient outcomes.

HDL is continuously remodeled in the bloodstream, resulting in a heterogeneous group of particles with diverse characteristics [7,8,9]. Traditionally considered a cholesterol transporter, HDL is now recognized for its multifaceted roles, encompassing cholesterol efflux capacity, antioxidant effects, anti-inflammatory actions, and immune-regulatory activities [7,10,11,12]. Small, dense HDL particles demonstrate a greater protective potential than larger particles, exhibiting enhanced cholesterol efflux capacity, stronger antioxidant properties, and more potent anti-inflammatory activities [13,14,15].

Although CKD patients have high cardiovascular risks, neither HDL-cholesterol levels nor HDL cholesterol efflux capacity predict events in this group [16]. Traditional cardiovascular risk factors only partially explain the increased disease incidence in CKD, as atherosclerosis may play a diminished role in advanced CKD. Kidney dysfunction is associated with changes in HDL maturation, in part through decreased lecithin cholesterol acyltransferase activity [17,18,19]. Subsequently, HDL levels are often reduced, and the lipoproteins exhibit structural and functional alterations [20,21,22,23]. These changes lead to the loss of key HDL properties, including its ability to promote cholesterol efflux from peripheral cells, as well as its vasodilatory, anti-inflammatory, and immunomodulatory activities [24,25,26,27,28,29]. Collectively, these alterations may contribute to the elevated cardiovascular and non-cardiovascular mortality observed in patients with CKD. As HDL particles vary in size and protein content, studying HDL subclass composition may help to clarify the link between HDL, cardiovascular disease, and high CKD-associated mortality.

In this study, we conducted a comprehensive analysis of HDL subclasses and their compositional characteristics to determine whether specific alterations in HDL subclasses are associated with adverse outcomes in CKD patients.

## 2. Materials and Methods

### 2.1. Study Design and Patients

The CARE FOR HOMe (Cardiovascular and Renal Outcome in CKD 2–4 Patients—The Fourth Homburg Evaluation) study is a prospective cohort study that originally enrolled 526 patients with CKD severity stages G2–G4, determined by the estimated glomerular filtration rate (eGFR) at baseline. Of these, 463 patients were included in our analysis based on the availability of sufficient serum material for performing lipoprotein profiling using NMR measurements. The study aimed to identify risk factors for CKD progression and to identify high-risk patients for cardiovascular complications. All participants received treatment at the renal outpatient clinic of the Saarland University Medical Centre in Homburg, Germany.

Exclusion criteria for the study included individuals with acute kidney injury, transplant recipients, pregnant women, those under 18 years of age, patients receiving systemic immunosuppressive medications, and patients with evident infections. Baseline fasting blood samples and a spot urine sample were collected from participants. A standardized questionnaire was administered to gather data on medical history, current medications, smoking status, and diabetes mellitus prevalence. The presence of cardiovascular disease was confirmed through a review of medical records, which included histories of coronary artery angioplasty, stenting, bypass surgery, myocardial infarction, carotid endarterectomy, stenting, non-traumatic lower extremity amputation, lower limb artery procedures, and major stroke.

Participants were invited to undergo annual follow-up examinations at the study center. The primary endpoint for these analyses was all-cause mortality, while additional endpoints had been described in previous studies [18,30].

### 2.2. Lipoprotein Profiling Using NMR Spectroscopy

Blood serum samples for NMR spectroscopy analysis were prepared as described previously [31,32]. The NMR spectra were obtained using a Bruker 600 MHz Avance Neo NMR spectrometer (Bruker, Rheinstetten, Germany). Lipoprotein quantification was performed using the Bruker IVDr Lipoprotein Subclass Analysis (B.I.LISA™) method. This process involves sending the raw NMR spectral data to a Bruker online server, which analyzes the data and returns 112 lipoprotein parameters. These parameters include parameters such as free and total cholesterol, triglyceride levels, and total phospholipid content across the main classes and subclasses of VLDL, IDL, LDL, and HDL, along with concentrations of proteins like ApoA-I, ApoA-II, and ApoB.

The Bruker IVDr Lipoprotein Subclass Analysis identifies four HDL subclasses (HDL-1 to HDL-4 sorted according to increasing density and decreasing size, respectively). The density ranges for these subclasses are defined as follows: HDL-1 ranges from 1.063 to 1.100 kg/L, HDL-2 from 1.100 to 1.112 kg/L, HDL-3 ranges from 1.112 to 1.125 kg/L, and HDL-4 from 1.125 to 1.210 kg/L. For simplicity, the subclasses have been labeled as L-HDL (HDL-1), M-HDL (HDL-2), S-HDL (HDL-3), and XS-HDL (HDL-4).

### 2.3. Statistical Analyses

Patients were stratified into two groups based on whether they reached the endpoint of all-cause mortality during the follow-up period. Clinical characteristics of the groups were compared using one-way ANOVA, the Wilcoxon rank sum test, and Pearson’s Chi-squared test and were summarized using mean (SD) for normally distributed variables and median (Q1, Q3) for variables with skewed distributions. Categorical variables are presented as the number of patients (percentage).

For Cox regression analyses, HDL subclass parameters were standardized, and hazard ratios were expressed per increase of 1 standard deviation (SD). Receiver operating characteristic (ROC) curve analysis was conducted to evaluate the prognostic ability of HDL-related parameters. A significance level of 0.05 was assumed. The statistical analysis was carried out using R version 4.4.1, Graphpad Prism version 10.2.2., and SPSS Statistics 26.

## 3. Results

### 3.1. Baseline Characteristics of the Study Cohort

In our study cohort, 463 CKD patients were included. The clinical characteristics are shown in Table 1. The mean age of all patients was 65 (12) years, and 39% were female. Most of the patients were within CKD stages 3a (32.6%) and 3b (28.1%). Among the patients, 33% had prevalent CVD, while the prevalence of diabetes mellitus was 39%. In Supplementary Table S1, the clinical characteristics of CKD patients stratified by CKD severity stage are presented.

We stratified patients of the CARE FOR HOMe study based on their survival status during the follow-up period. During a mean follow-up time of 5.0 ± 2.2 years, 86 patients (18.6%) reached the endpoint of all-cause mortality. Deceased patients were significantly older (72 (9) vs. 63 (12), *p* < 0.001) and predominantly male (74% vs. 58%, *p* = 0.005). The groups also differed in baseline eGFR, CRP levels, smoking status, and diastolic blood pressure. Additionally, the prevalence of CVD and diabetes mellitus was higher in the patients who died, with both conditions affecting more than fifty percent of this group. There were no significant differences in the use of statins or other lipid-lowering medications between the two groups. With a median BMI of 30.1 (26.8–33.4), the majority of patients were overweight or obese. However, there were no significant differences in BMI between patients who survived and those who were deceased. Both LDL- and HDL-cholesterol levels were lower in patients who reached the endpoint, which also corresponded to reduced levels of plasma total cholesterol. There was no significant difference in baseline triglyceride levels between patient groups.

### 3.2. HDL Subclass Parameters from Lipoprotein Profiling Using NMR Spectroscopy in CKD Patients

**Figure 1:**
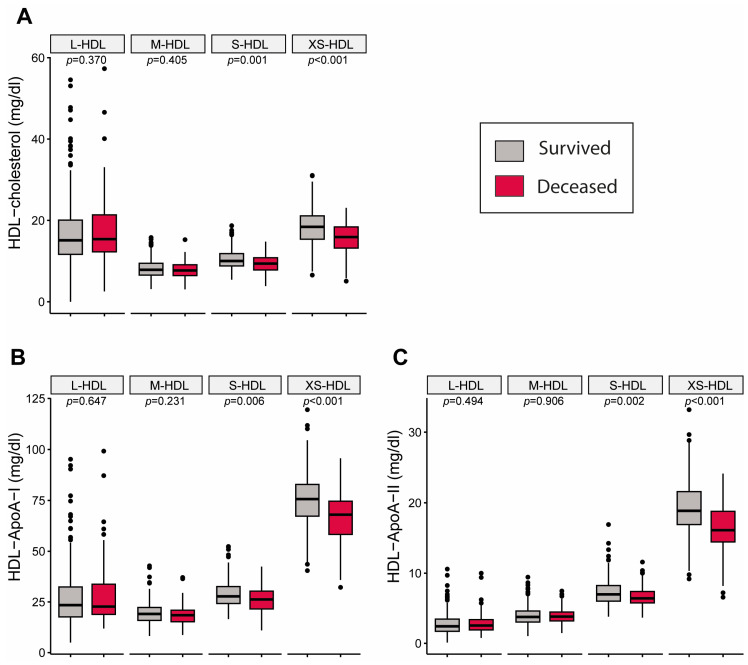
Differences in baseline HDL-subclasses among CKD patients, categorized by survival during the follow-up period. The endpoint was defined as all-cause mortality. (**A**) depicts variations in HDL-associated cholesterol levels among subclasses, while (**B**,**C**) illustrates the respective ApoA-I and ApoA-II levels. Statistical differences between the groups were evaluated using the Wilcoxon rank sum test. Data are displayed as Tukey boxplots, with outliers indicated by black dots. Apo, Apolipoproteins.

Lipoprotein profiling by NMR spectroscopy allows the analysis of compositional parameters within HDL subclasses, ranging from large (L-) to extra-small (XS-) HDL. We observed significant differences in the cholesterol content of S- and XS-HDL particles (Figure 1). In addition, the levels of ApoA-I and ApoA-II were also reduced in S- and XS-HDL-subclasses in patients who died during the follow-up period.

### 3.3. HDL-Related Parameters and the Risk of Mortality in Patients with CKD

**Figure 2:**
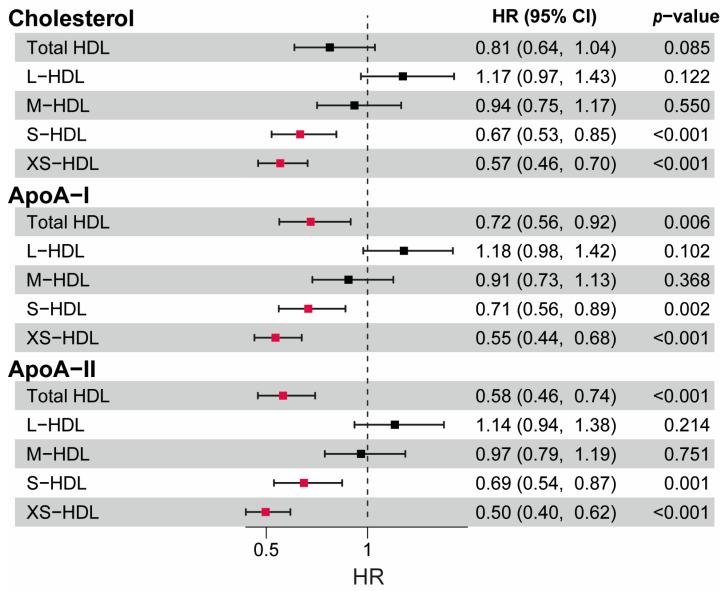
Cox-regression analyses of HDL-related parameters with all-cause mortality. Hazard ratios (HR) per 1 standard deviation (SD) increase. Significant associations are highlighted in red.

To examine the associations between HDL subclass parameters and mortality risk in CKD, we conducted univariable Cox regression analyses on the evaluated HDL subclass parameters (Figure 2). Notably, parameters of the S- and XS-HDL subclasses demonstrated a strong inverse association with mortality risk, with XS-HDL-ApoA-II exhibiting the most significant association (HR 0.50 per 1 SD increase, 95% CI 0.40, 0.62, *p* < 0.001). Total HDL-ApoA-I and HDL-ApoA-II levels were also inversely associated with the risk of all-cause mortality.

To assess these associations independently of other renal and cardiovascular risk factors, we performed multivariable Cox regression analyses on all parameters that showed significance in the univariable model, adjusting for multiple confounders (Table 2). In the first model, we adjusted for age, gender, BMI, and eGFR. Most parameters that were significantly associated with mortality risk in the univariable analyses remained significant after adjustment, except for total HDL-ApoA-I. After further adjustment for additional cardiovascular risk factors in Model 2, only the compositional parameters of the XS-HDL subclass—specifically cholesterol; ApoA-I; and ApoA-II—continued to demonstrate a significant inverse association with mortality risk. The strongest inverse association was observed for XS-HDL-ApoA-II (HR 0.69 per 1 SD increase, 95% CI 0.53–0.88, *p* = 0.003).

The association of XS-HDL parameters with other available endpoints of this study is shown in Supplementary Table S2.

**Figure 3:**
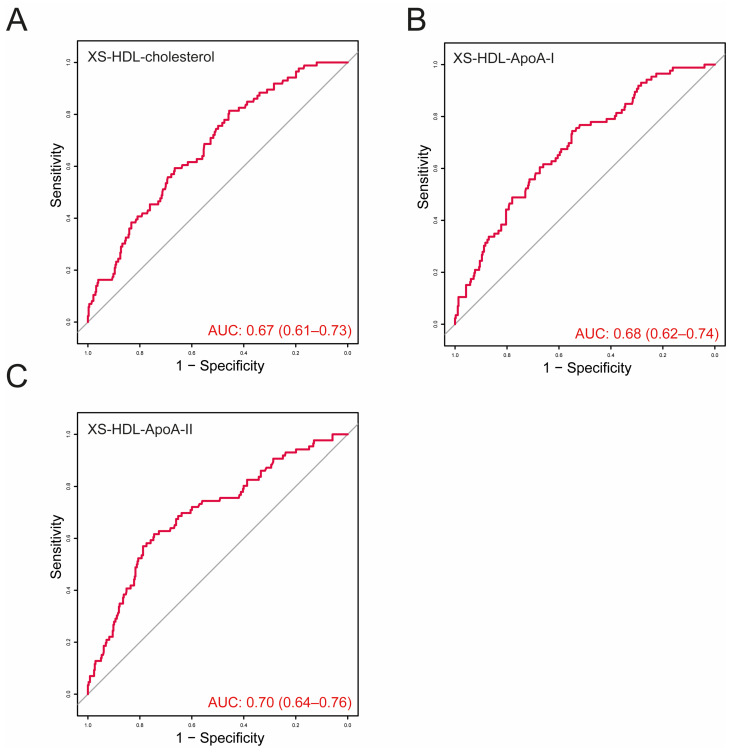
Receiver operating characteristic (ROC) curves for predicting all-cause mortality in CKD patients based on (**A**) cholesterol, (**B**) ApoA-I, and (**C**) ApoA-II levels within the XS-HDL subclass.

To evaluate the prognostic value of the significant variables identified in the Cox regression analyses for all-cause mortality in CKD patients, we conducted receiver operating characteristic (ROC) curve analyses (Figure 3). Notably, XS-HDL-ApoA-II exhibited the highest predictive potential, with an area under the curve (AUC) of 0.70 (0.64–0.76).

In the present study cohort, 44 patients died as a result of a confirmed cardiovascular complication. However, when the causes of death were analyzed separately, no significant association was observed between XS-HDL parameters and cardiovascular mortality (Table 3).

## 4. Discussion

The prevalence of CKD is increasing, making it the seventh leading cause of death worldwide [33,34]. This trend underscores the critical need for early detection and preventive strategies. While established biomarkers and predictive models are available to assess outcomes in CKD patients, accurate estimation remains challenging and is frequently associated with poor patient outcomes [35,36,37].

To our knowledge, this is the first study to investigate the relationship between HDL subclasses and mortality risk in CKD patients. We identified a robust association between low plasma levels of the XS-HDL subclass, particularly XS-HDL containing ApoA-II, and increased mortality risk. Notably, this association persisted even after adjusting for conventional cardiovascular and renal risk factors. In contrast, total HDL-cholesterol, total HDL-ApoA-I, and total HDL-ApoA-II were not independently associated with mortality; only the parameters of the small subclasses were linked to mortality risk. It is important to note that key biological activities of HDL subpopulations, including cholesterol efflux capacity, antioxidative activity against low-density lipoprotein oxidation, anti-inflammatory activity, and antiapoptotic activity in endothelial cells, are primarily associated with small, dense, protein-rich HDL particles. Particularly small, dense, protein-rich HDL may offer significant protection against oxidative damage from free radicals in the arterial intima, thereby inhibiting the formation of proinflammatory oxidized lipids [38].

In CKD, HDL particle maturation is impaired, as evidenced by reduced lecithin-cholesterol acyltransferase activity and compromised renal clearance of immature HDL particles [17,18,21,39]. This dysfunction significantly delays the conversion of pre-β-1 HDL to α-migrating HDL, leading to an accumulation of pre-β-1 particles [39]. We observed that the S- and XS-HDL subclasses were most strongly inversely associated with mortality risk in CKD patients. Notably, XS-HDL-ApoA-II demonstrated the strongest association, correlating with a 31% reduction in mortality risk for each 1-SD increase. ROC curve analyses further supported the prognostic value of XS-HDL-ApoA-II, with an AUC of 0.70.

In alignment with our findings, higher levels of total ApoA-II, rather than ApoA-I, have been associated with a lower risk of all-cause mortality in the German Diabetes Dialysis study [40]. Moreover, both ApoA-II and small HDL subclasses have been shown to predict mortality in patients with acute heart failure [32,41]. These findings suggest that the strong association between ApoA-II and reduced mortality risk may reflect a protective role of this protein.

Interestingly, when we analyzed causes of death separately, we found no significant association between XS-HDL parameters and cardiovascular mortality. This lack of association may be due to a shift in the type of CVD experienced by these patients, with a greater prevalence of non-atherosclerotic forms that are not responsive to alterations in lipoprotein metabolism. It is also notable that a substantial proportion of CKD patients die from non-cardiovascular causes, such as infections [42]. As a naturally occurring nanoparticle, HDL is capable of binding and neutralizing a range of harmful substances, including bacterial lipopolysaccharides. Furthermore, HDL has been demonstrated to possess antiviral properties, which inhibit viral invasion and replication [43]. Consistent with this hypothesis, low levels of small HDL particles have been linked to higher infectious disease mortality rates, such as sepsis [44]. Notably, ApoA-II may play a role in promoting the formation of these small HDL particles [45,46,47,48]. Recent evidence suggests that HDL particles containing both ApoA-I and ApoA-II have a wider range of particle sizes than those containing only ApoA-I, suggesting that ApoA-II is a major factor in HDL size heterogeneity [49]. Consequently, further research on ApoA-II-containing HDL particles is required. Furthermore, mass spectrometry analysis may potentially identify specific components of XS-HDL with enhanced prognostic value [50]. Specifically, the HDL lipidome significantly influences the biological function of HDL particles. In particular, the content of phosphatidylserine in small, dense HDL3 was observed to have positive correlations with metrics of HDL functionality [51].

While our study focused on prospective clinical outcomes, previous research on HDL particle size in CKD patients has primarily examined associations with prevalent cardiovascular disease [52,53]. For example, a recent study using ion mobility analysis (IMA) found that low levels of medium-sized HDL particles were associated with incident CVD, while other subclasses showed no association [53]. Another study using IMA in patients with type 1 diabetes found that XS HDL particle concentration was the strongest inverse predictor of coronary heart disease [54]. It is important to note that methodological differences between NMR and IMA to measure HDL subclasses differently may make it difficult to directly compare the results. NMR focuses on particle size distribution, whereas IMA assesses both size and charge.

We acknowledge some limitations of this study. The moderate sample size (n = 463) may have limited our ability to detect subtle differences, especially considering the diverse underlying conditions contributing to CKD. Moreover, mass spectrometry analysis of XS-HDL subclasses could potentially reveal further components of XS-HDL with enhanced prognostic value. Furthermore, the observational design precludes establishing definitive causal relationships.

A key strength of our study is its prospective design with a long follow-up period, allowing for a robust assessment of outcomes over time. Additionally, comprehensive baseline clinical characterization enabled effective control for potential confounding factors. Our findings prompt further exploration into the broader role of HDL beyond cardiovascular events, with the potential to uncover novel therapeutic strategies.

In conclusion, this prospective cohort study is the first to show that low levels of XS-HDL are strongly associated with an increased risk of all-cause mortality in CKD patients. Unlike total HDL-cholesterol, these XS-HDL markers offer significantly greater prognostic value, highlighting their potential as valuable tools for mortality risk prediction in this population.

## Figures and Tables

**Table 1:** Baseline clinical characteristics of the study cohort.

Characteristic	Survived (n = 377)	Deceased (n = 86)	All (n = 463)	*p*-Value
Age (years)	63 (12)	72 (9)	65 (12)	<0.001 ^1^
Sex, Female (n, %)	158 (42%)	22 (26%)	180 (39%)	0.005 ^2^
BMI (kg/m^2^)	30.3 (26.9, 33.4)	29.6 (26.3–33.7)	30.1 (26.8, 33.4)	0.533 ^3^
eGFR (ml/min/m^2^)	48.8 (37.2, 59.)	33.6 (25.5, 45.0)	46.5 (33.7, 57.5)	<0.001 ^3^
CKD severity stages				
CKD stage G2 (%)	92 (24.4%)	6 (7.0%)	98 (21.2%)	<0.001 ^2^
CKD stage G3a (%)	136 (36.1%)	15 (17.4%)	151 (32.6%)	<0.001 ^2^
CKD stage G3b (%)	96 (25.5%)	34 (39.5%)	130 (28.1%)	0.009 ^2^
CKD stage G4 (%)	53 (14.1%)	31 (36.0%)	84 (18.1%)	<0.001 ^2^
CRP (mg/L)	2.4 (1.1, 4.5)	4.0 (1.8, 7.7)	2.7 (1.1, 5.0)	<0.001 ^3^
Hba1c (%)	5.8 (5.5, 6.4)	5.8 (5.6, 7.1)	5.8 (5.5, 6.4)	0.559 ^3^
GOT (U/L)	26 (22, 31)	25 (21, 31)	26 (22, 31)	0.606 ^3^
Prevalent CVD (n, %)	101 (27%)	50 (58%)	151 (33%)	<0.001 ^2^
Diabetes Mellitus (n, %)	133 (35%)	47 (55%)	180 (39%)	<0.001 ^2^
Current smoking (n, %)	49 (13%)	3 (4%)	52 (11%)	0.012 ^2^
Statins (n, %)	192 (51%)	48 (56%)	240 (52%)	0.413 ^2^
Other lipid-lowering drugs (n, %)	46 (12%)	8 (9%)	54 (12%)	0.450 ^2^
Systolic BP (mmHg)	149 (137, 166)	154 (139, 173)	149 (137, 167)	0.129 ^3^
Diastolic BP (mmHg)	86 (77, 95)	82 (72, 89)	85 (76, 94)	0.002 ^3^
Total cholesterol (mg/dL)	196 (167,228)	182 (154, 215)	192 (165, 224)	0.007 ^3^
Triglycerides (mg/dL)	150 (109, 212)	144 (107, 210)	149 (109, 211)	0.433 ^3^
LDL-cholesterol (mg/dL)	93 (75, 116)	86 (61, 100)	92 (72, 114)	0.004 ^3^
HDL-cholesterol (mg/dL)	52 (45, 61)	49 (42, 57)	51 (45, 61)	0.030 ^3^

Differences between the groups were calculated with ^1^ One-way ANOVA, ^2^ Pearson’s Chi-squared test, or ^3^ Wilcoxon rank sum test. Values for categorical variables are given as numbers (percentages), and values for continuous variables are given as mean (SD) or median (Q1–Q3). BMI, body mass index; eGFR, estimated glomerular filtration rate; CRP, C-reactive protein; GOT, glutamic-oxaloacetic transaminase; CVD, cardiovascular disease; BP, blood pressure.

**Table 2:** Multivariable Cox-regression analyses of HDL-related parameters with risk of death in CKD.

	Model 1		Model 2	
Parameter	HR (95% CI) Per 1 SD	*p*-Value	HR (95% CI) Per 1 SD	*p*-Value
Total HDL-cholesterol	0.97 (0.75, 1.26)	0.827	1.10 (0.86, 1.42)	0.457
S-HDL-cholesterol	0.75 (0.58, 0.96)	0.019	0.89 (0.69, 1.16)	0.376
XS-HDL-cholesterol	0.67 (0.53, 0.84)	<0.001	0.77 (0.60, 0.98)	0.031
Total HDL-ApoA-I	0.83 (0.64, 1.08)	0.165	1.01 (0.78, 1.30)	0.940
S-HDL-ApoA-I	0.77 (0.60, 0.99)	0.032	0.92 (0.71, 1.18)	0.502
XS-HDL-ApoA-I	0.65 (0.52, 0.81)	<0.001	0.76 (0.60, 0.97)	0.028
Total HDL-ApoA-II	0.69 (0.54, 0.88)	0.002	0.83 (0.64, 1.07)	0.144
S-HDL-ApoA-II	0.75 (0.59, 0.96)	0.016	0.87 (0.68, 1.11)	0.254
XS-HDL-ApoA-II	0.59 (0.47, 0.74)	<0.001	0.69 (0.53, 0.88)	0.003

Cox-regression analyses of HDL subclasses with risk of all-cause mortality. Model 1 was adjusted for Age, Sex, BMI, and eGFR. Model 2 is Model 1 additionally adjusted for prevalent CVD, diabetes mellitus, systolic and diastolic blood pressure, current smoking, log-transferred CRP, statin medication, and other lipid-lowering drugs.

**Table 3:** Multivariable Cox-regression analyses of HDL-subclass parameters with CVD-related mortality risk.

	CVD Mortality (n = 44)	
Parameter	HR (95% CI) Per 1 SD	*p*-Value
XS-HDL-cholesterol	0.82 (0.57, 1.18)	0.288
XS-HDL-ApoA-I	0.79 (0.56, 1.14)	0.208
XS-HDL-ApoA-II	0.79 (0.56, 1.11)	0.172

Cox-regression analyses of HDL subclasses with risk of CVD mortality. The model is adjusted for age, sex, BMI, eGFR, prevalent CVD, diabetes mellitus, systolic and diastolic blood pressure, current smoking, log-transferred CRP, statin medication, and other lipid-lowering drugs.

## Data Availability

Data are contained within the article and Supplementary Material.

## Supplementary Materials

The following supporting information can be downloaded at: https://www.mdpi.com/article/10.3390/antiox13121511/s1; Table S1: Clinical characteristics of CKD patients stratified by CKD severity stage. Table S2: Cox-regression analyses of XS-HDL parameters with clinical endpoints in CKD.
