# Supplementary material for: Association of Small HDL Subclasses with Mortality Risk in Chronic Kidney Disease
Source: Antioxidants (Basel). 2024 Dec 11;13(12):1511. doi: 10.3390/antiox13121511 (PMC11673888; doi:10.3390/antiox13121511)
Supplement: Supplementary file 1 [file antioxidants-13-01511-s001.zip › antioxidants-3340957-supplementary.pdf]

Supplementary Material:

Suppl. Table S1. Clinical characteristics of the study cohort stratified by CKD severity stages.

Characteristic	Overall, N = 463[1]	G 2, N = 98[1]	G 3 a, N = 151[1]	G 3 b, N = 130[1]	G 4, N = 84[1]	p-value
**Age (y)**	65 (12)	59 (12)	65 (13)	68 (11)	68 (12)	**<0.001**[1]
**Female sex**	180 (38.9%)	30 (30.6%)	63 (41.7%)	57 (43.8%)	30 (35.7%)	0.169[3]
**BMI (kg/m2)**	30.1 (26.8,33.4)	29.7 (26.5,33.5)	30.5 (27.0,33.7)	30.4 (26.5,33.2)	28.9 (26.6,32.9)	0.503[3]
**eGFR (ml/min/m2)**	47 (34,58)	67 (63,73)	52 (48,55)	37 (34,41)	23 (19,26)	**<0.001**[4]
**CRP (mg/l)**	2.7 (1.2,5.0)	2.3 (1.2,3.9)	2.5 (1.1,4.9)	2.9 (1.2,6.2)	3.4 (1.1,5.7)	0.185[3]
**Hba1c (%)**	5.80 (5.50,6.40)	5.60 (5.40,6.00)	5.70 (5.50,6.38)	6.10 (5.60,6.90)	6.00 (5.60,6.33)	0.454[3]
**GOT (U/l)**	26 (22,31)	26 (22,32)	25 (23,31)	26 (22,32)	24 (19,28)	**0.049**[3]
**Prevalent CVD (n, %)**	151 (32.6%)	17 (17.3%)	48 (31.8%)	58 (44.6%)	28 (33.3%)	**<0.001**[2]
**Diabetes Mellitus (n, %)**	180 (38.9%)	33 (33.7%)	59 (39.1%)	50 (38.5%)	38 (45.2%)	0.465[2]
**Current nicotine (n, %)**	52 (11.2%)	19 (19.4%)	14 (9.27%)	12 (9.23%)	7 (8.33%)	**0.039**[2]
**Statins (n, %)**	240 (51.8%)	38 (38.8%)	83 (55.0%)	76 (58.5%)	43 (51.2%)	**0.022**[2]
**Other lipid lowering drugs (n,**	54 (11.7%)	14 (14.3%)	15 (9.93%)	18 (13.8%)	7 (8.33%)	0.458[2]
**Systolic BP (mmHg)**	149 (137,166)	145 (136,160)	153 (140,170)	146 (135,165)	152 (141,166)	0.055[3]
**Diastolic BP (mmHg)**	85 (76,94)	86 (79,96)	88 (79,96)	81 (72,90)	86 (74,94)	**0.001**[3]
**Total cholesterol (mg/dl)**	192 (165,224)	202 (179,230)	191 (163,226)	187 (163,216)	186 (160,218)	0.130[3]
**HDL-cholesterol (mg/dl)**	51 (45,61)	53 (45,62)	51 (45,61)	52 (45,61)	49 (41,59)	0.200[4]

**LDL-cholesterol (mg/dl)**	92 (72,114)	101 (79,121)	91 (74,109)	90 (73,106)	87 (65,110)	**0.023**[3]
**Triglycerides (mg/dl)**	149 (109,211)	146 (102,212)	149 (108,211)	150 (112,203)	155 (120,218)	0.764[3]

Differences between the groups were calculated with [1]One-way ANOVA, [2]Pearson's Chi-squared test or [3]Kruskal-Wallis test. Values for categorical variables are given as numbers (percentages) and values for continuous variables are given as mean (SD) or median (Q1-Q3). BMI, body mass index; eGFR, estimated glomerular filtration rate; CRP, C-reactive protein; GOT, glutamic-oxaloacetic transaminase; CVD, cardiovascular disease; BP, blood pressure.

Suppl. Table S2. Cox-regression analyses of XS-HDL parameters with clinical endpoints in CKD.

	Model 1		Model 2	
Parameter	HR (95% CI) Per 1 SD	*p*-value	HR (95% CI) Per 1 SD	*p*-value
MACE				
XS-HDL-cholesterol	0.78 (0.66, 0.93)	**0.007**	0.93 (0.76, 1.14)	0.495
XS-HDL-ApoA-I	0.73 (0.62, 0.87)	**<0.001**	0.89 (0.73, 1.08)	0.226
XS-HDL-ApoA-II	0.73 (0.61, 0.87)	**<0.001**	0.87 (0.71, 1.06)	0.155
Renal Decline				
XS-HDL-cholesterol	0.95 (0.75, 1.21)	0.693	1.06 (0.81, 1.38)	0.676
XS-HDL-ApoA-I	0.95 (0.75, 1.20)	0.673	1.07 (0.81, 1.39)	0.646
XS-HDL-ApoA-II	0.81 (0.64, 1.03)	0.078	0.89 (0.68, 1.17)	0.395
Acute decompensated heart failure				
XS-HDL-cholesterol	0.83 (0.62, 1.11)	0.206	0.91 (0.67, 1.24)	0.549
XS-HDL-ApoA-I	0.80 (0.60, 1.05)	0.110	0.89 (0.65, 1.21)	0.454
XS-HDL-ApoA-II	0.81 (0.60, 1.09)	0.158	0.90 (0.64, 1.25)	0.517

Cox-regression analyses of parameters of XS-HDL subclass with risk of MACE, renal decline and acute decompensated heart failure. Model 1 was adjusted for Age, Sex, BMI and eGFR. Model 2 is Model 1 additionally adjusted for prevalent CVD, diabetes mellitus, systolic and diastolic blood pressure, current smoking, log-transferred CRP, statin medication, and other lipid-lowering drugs. MACE, major atherosclerotic cardiovascular events including all-cause death.
